# Ethics approval: responsibilities of journal editors, authors and research ethics committees

**DOI:** 10.11604/pamj.2017.28.200.14170

**Published:** 2017-11-03

**Authors:** Luchuo Engelbert Bain

**Affiliations:** 1 Athena Institute for Research on Communication and Innovation in Health and Life Sciences, Vrije Universiteit, Amsterdam, The Netherlands

**Keywords:** Ethics approval, research ethics committees, researcher, journal, reviewers

## Abstract

Meaningful progress of medicine depends on research that must ultimately involve human subjects. Obtaining ethical approval therefore, especially in medical sciences, should be a moral reflex for researchers. This unfortunately is not the case, with numerous researchers bypassing the ethics approval procedure, or simply unaware of its importance. Good research involves risks taken by research participants and uses tax payers’ money in the process. These mandates the research endeavor to aim at attaining the highest degree of respect for the sacrifices made by others for science. Most researchers mistake scientific clearance or approval, for ethics approval. For a study to be ethical sound, it must be scientifically sound. This is only one of the activities carried out during protocol review. It is not uncommon for sensitive ethical concerns, especially in the social sciences to be overlooked and considered not to be accompanied by any serious risks for the research participants.The researcher has the responsibility of systematically consulting the competent ethics committee for advice and consequent approvals or ethical waivers. Journal editors and reviewers have the duty to systematically evaluate the ethical soundness of manuscripts submitted for review. Capacity building in research ethics and institutional support for Research Ethics Committees to speed up protocol review could reduce the incentive of carrying out research in human subjects without ethics approvals. It is hypocritical and idle to continue to expect optimal reviews on time and of good quality, from ethics committees functioning purely on altruistic grounds. Capacity building for researchers in research ethics, and institutional reforms and support for Research Ethics Committees appear not to have received the attention they truly deserve.

## Editorial

Meaningful progress of medicine depends on research that must ultimately involve human subjects [1]. The researcher carries the responsibility to respect the highest ethical standards to protect research participants even after consent is obtained [1]. The amount of research on human subjects inundating the world is taking geometric proportions and the trend is almost irreversible. The Declaration of Helsinki requires that all medical researches be submitted to and approved by an ethics committee. It states: “*the research protocol must be submitted for consideration, comment, guidance and approval to a research ethics committee before the study begins*” [1]. Obtaining ethical approval therefore, especially in medical sciences, should be a moral reflex for researchers. Good research involves risks taken by research participants and most of the time uses tax payers' money in the process. These mandates the research endeavor to aim at attaining the highest degree of respect for the sacrifices made by others for science. How can the young researcher respect ethical standards, without any prior research ethics knowledge or training, not to talk of knowing what an ethics approval is all about? Of 174 theses on Human Immuno Virus (HIV) in Cameroon, only 17 out of these had documented ethics approvals [2]. Out of 217 reviewed full length articles in the health sciences, 57.53% of these reported having obtained ethical approval [3]. Seven items generally constitute the research ethics review agenda for most protocols [4]. Social value; scientific validity; fair subject selection; independent review; informed consent; respect for enrolled subjects; favorable risk-benefit ratio. Most researchers mistake scientific clearance or approval for ethical clearance. For a study to be ethical sound, it must be scientifically sound. This is only one of the activities carried out during protocol review. Most Research Ethics Committees (RECs), or elsewhere, Institutional Review Boards (IRBs) are transdisciplinary in constitution to properly ascertain the diverse dimensions of the research question under investigation. This reduces the bias of looking at the “black bird” from a single direction. It is not uncommon for sensitive ethical concerns, especially in the social sciences to be overlooked and considered not to be accompanied by any serious risks of the research participants. It is important to highlight the fact that risk is not only physical, but certain exposures to the research task might have far reaching psychosocial consequences. In laboratory, genetic medicine, biobank research and disaster response, core ethical concerns seen in broad consent might be erroneously omitted at the beginning of the study, or fail to be sought from participants for instance during initial sample collection phase. RECs could be helpful to highlight these issues to the research team. Obtaining ethics approval is a core component of good science. Publication pressure is a key contributor to falsification of research findings, as well as bypassing ethics approval, especially among young researchers. Failure to properly ascertain the importance of ethical approval by researchers and journals could indirectly fuel research misconduct and predator publication [5, 6]. Researchers could shy away from submitting ethically charged protocols for review, or go for “low quality or less stringent” RECs. Complexities of the protocol review process in terms of money, time and requirements could scare researchers from engaging into the endeavor to obtain ethical approval [7]. With the already worrisome publish or perish syndrome, low quality ethics review, or complete avoidance and consequent opting for predator journals becomes the way out for these researchers. Many, if not all respected journals require an ethics statement. The editors and reviewers routinely check if ethical approval was obtained for research on human subjects.

Though a duty for the reviewers to be sensitive to the ethics of the manuscript under consideration, it is the sole responsibility of the author or researcher to ensure that he obtains ethics approval if need be. Obtaining ethics approval does not in itself render the research work ethical. Competence of the RECs to review specific types of research need to be continuously questioned and defined by the respective National Ethics Committees (NECs). Capacity building in research ethics should remain a priority. Researchers must themselves recognize the importance of obtaining ethical clearance. In cases of doubt whether to obtain approval or not, advice from the ethics committee, research ethics specialists should be systematically sought. The future might compel the creation of national and international data bases of RECs, for rapid cross checking of the validity of provided ethical approval references or copies provided by authors. Many authors could declare having obtained ethical approval, while in practice, no ethical approval in effect was actually obtained. It is a subject of discussion to systematically provide references or copies of the ethical approvals to journal editorial boards to be sure these were obtained. NECs have the responsibility to have a data base of smaller RECs, define their scope of competence and ensure capacity building [7]. Lack of coordination could make ethically charged researchers to go to small inexperienced RECs for more rapid approvals. Training and capacity building remains a key challenge, and students must receive compulsory research ethics courses as researchers of tomorrow. It is only in this light that they shall develop the moral responsibility as researchers to systematically reflect on obtaining ethical approvals before delving into the research process. Most researchers and students erroneously self-evaluate specific studies not to be ethically charged and find ethical approval an idle venture. However, the ethical waiver decision or advice preferably should come from the ethics committee. The challenges for RECs remain persistent.

Most functioning on altruistic grounds, hold meetings once in a month that delay the review process, and thus scare scientists from seeking ethical approval. Governments must support the functioning and encourage capacity building of these ethics committees. Research malpractice is here and it shall be scientific hypocrisy to think this could be eliminated by the good will of researchers. Ethical “policing” through requests of ethical approval references or copies for eventual verification should be considered. Should it be a mandatory task for reviewers to ask for copies/references of ethical approvals before going on to publication of research involving human subjects? Should reviewers trust the declarations of scientists on grounds of mere ethics statements? How can the validity of assertions of having obtained ethical approval be verified? Is it the duty/role of the reviewer to check if ethics approval was effectively obtained? It is time for NECs to think of National REC repositories, where authenticity of ethics approvals could be verified. This could be helpful in case of allegations of unethical research or exploitation that could potentially arise. Verification of ethics approval authenticity could serve as a nudge to promote ethical sound research. Obtaining ethical approval is above all, the responsibility of the author or researcher. Reviewers have a mandate to verify these ethics statements. Research ethics education and capacity building remain key action areas that have not received the attention they righty deserve in the past. Including ethics statements in reports could encourage researchers to seek for ethics approval, and thus carryout ethical research [8]. This statement on its own, is simply a means, and not an end itself. Ethical approval must become part of researchers? daily practice and requires to be systematically checked by reviewers before publication of research involving human subjects. A discussion on whether or not to verify the authenticity of provided ethics approval references or copies, as well as the establishment of Research Ethics Committee Repositories at National and International Levels looks promising. This could indirectly facilitate collaborative research, as well as arouse the need to systematically obtain ethics approval among researchers. Institutional support to speed up protocol review could reduce the incentive of carrying out research in human subjects without ethics approval.

## Competing interests

The author declare no competing interest.
